# Prevalence of diabetic retinopathy in Brazil: a systematic review with meta-analysis

**DOI:** 10.1186/s13098-023-01003-2

**Published:** 2023-03-02

**Authors:** Thiago Alves Chagas, Mateus Augusto dos Reis, Gabriel Leivas, Lucas Porto Santos, Agnes Nogueira Gossenheimer, Gustavo Barreto Melo, Fernando Korn Malerbi, Beatriz D. Schaan

**Affiliations:** 1 Instituto de Olhos Cristiano Mendonça, Aracaju, SE Brazil; 2 Graduate Program in Medical Sciences: Endocrinology, Universidade Federal do Rio Grande do Sul, Porto Alegre, RS Brazil; 3 National Institute of Science and Technology for Health Technology Assessment (IATS), Porto Alegre, RS Brazil; 4 Department of Ophthalmology and Visual Science, Universidade Federal de São Paulo, São Paulo, SP Brazil; 5 Endocrine Division, Hospital de Clínicas de Porto Alegre, Porto Alegre, RS Brazil

**Keywords:** Diabetic retinopathy, Diabetes mellitus, Diabetes complications

## Abstract

**Aims:**

To evaluate the prevalence of diabetic retinopathy (DR) in Brazilian adults with diabetes mellitus via a systematic review with meta-analysis.

**Methods:**

A systematic review using PubMed, EMBASE, and Lilacs was conducted, searching for studies published up to February 2022. Random effect meta-analysis was performed to estimate the DR prevalence.

**Results:**

We included 72 studies (n = 29,527 individuals). Among individuals with diabetes in Brazil, DR prevalence was 36.28% (95% CI 32.66–39.97, I^2^ 98%). Diabetic retinopathy prevalence was highest in patients with longer duration of diabetes and in patients from Southern Brazil.

**Conclusion:**

This review shows a similar prevalence of DR as compared to other low- and middle-income countries. However, the high heterogeneity observed—expected in systematic reviews of prevalence—raises concerns about the interpretation of these results, suggesting the need for multicenter studies with representative samples and standardized methodology.

**Supplementary Information:**

The online version contains supplementary material available at 10.1186/s13098-023-01003-2.

## Introduction

Diabetes mellitus is a metabolic disease that may lead to chronic microvascular and macrovascular complications [1]. Diabetic retinopathy (DR)—the most common complication of diabetes mellitus—is one of the leading causes of preventable blindness in the adult population [2]. Vision impairment and blindness due to diabetes may be irreversible if timely treatment is not provided, affecting the individual’s functional capabilities and self-care [3]. Moreover, DR is considered a risk factor for other diabetes complications [4].

The International Diabetes Federation estimates that 537 million adults live with diabetes in 2021 [5]. In Brazil, a systematic review estimated a 6.9% prevalence of diabetes in the population based on studies published after 2010 [6]. With an aging population, coupled with growing rates of diabetes, a higher burden of DR and demand for eye care and treatment are expected [2]. The international literature on DR epidemiology has several population studies, such as the WESDR [7], UKPDS [8], DCCT [9], and ETDRS [10], and a recent systematic review by Teo et al. has concluded that—amongst individuals with diabetes—the global prevalence of DR is estimated at 22.27% [2]. However, factors such as varying levels of surveillance, different socio-economic factors, and health systems organization can prompt differences in estimated DR prevalence among countries [11–14].

Brazil is a large upper-middle-income country that hosts the world’s sixth largest population of individuals with diabetes [15]; it is also the country with the largest free public health care system [16], on which around 75% of its population relies [17]. National data on the prevalence of DR are lacking, but regional studies indicate a prevalence ranging from 7.6 to 44.4% of individuals with diabetes, with great regional and methodological variations in each survey [18–23].

The diagnosis of DR comprises the detection of ophthalmological lesions in ophthalmoscopy or color fundus photographs that are considering in classifying DR. The classification defines the prognosis and the need for treatment. More advanced degrees of DR have a worse prognosis. DR is classified as proliferative and non-proliferative, being divided into mild, moderate and severe; macular edema may or may not be present [24].

Because DR is a major public health issue, demanding thoughtful resource allocation, and since blindness is preventable with timely treatment, planning from health authorities is crucial. Since no national strategies or standardized workflows for DR screening and management in the Brazilian public health system currently exist [25], estimating the prevalence of DR and its regional variations is a crucial step for designing such policies and for an effective resource and workforce allocation. This study aims to assess the prevalence of DR in Brazil; additionally, this study aims to evaluate other aspects of DR epidemiology, such as geographic differences and risk factors.

## Methods

This report describes a systematic review and meta-analysis of studies describing the DR prevalence in individuals with diabetes in Brazil. All procedures herein described were conducted in accordance with the Preferred Reporting Items for Systematic Reviews and Meta-Analyses and Meta-analyses Of Observational Studies in Epidemiology guidelines [26]. The protocol for this review was registered and publicly available at PROSPERO (CRD42022362777).

### Search strategy

Three databases (PubMed, LILACS, and EMBASE) were systematically searched using terms related to diabetes, retinopathy, and prevalence. Papers written in English, Portuguese, or Spanish were retrieved, from inception to February 2022. The detailed search strategy can be consulted in Additional file 1: Table S1.

### Eligibility criteria

Articles meeting the following criteria were included: (1) designed as cross-sectional, cohort, or case–control studies, (2) conducted in Brazil, (3) describing the frequency of adults with DR among those with type 1 diabetes or type 2 diabetes. Studies including pregnant women, patients with diabetes other than type 1 or type 2 diabetes mellitus, or conducted outside Brazil were excluded.

### Study selection

**Fig. 1:**
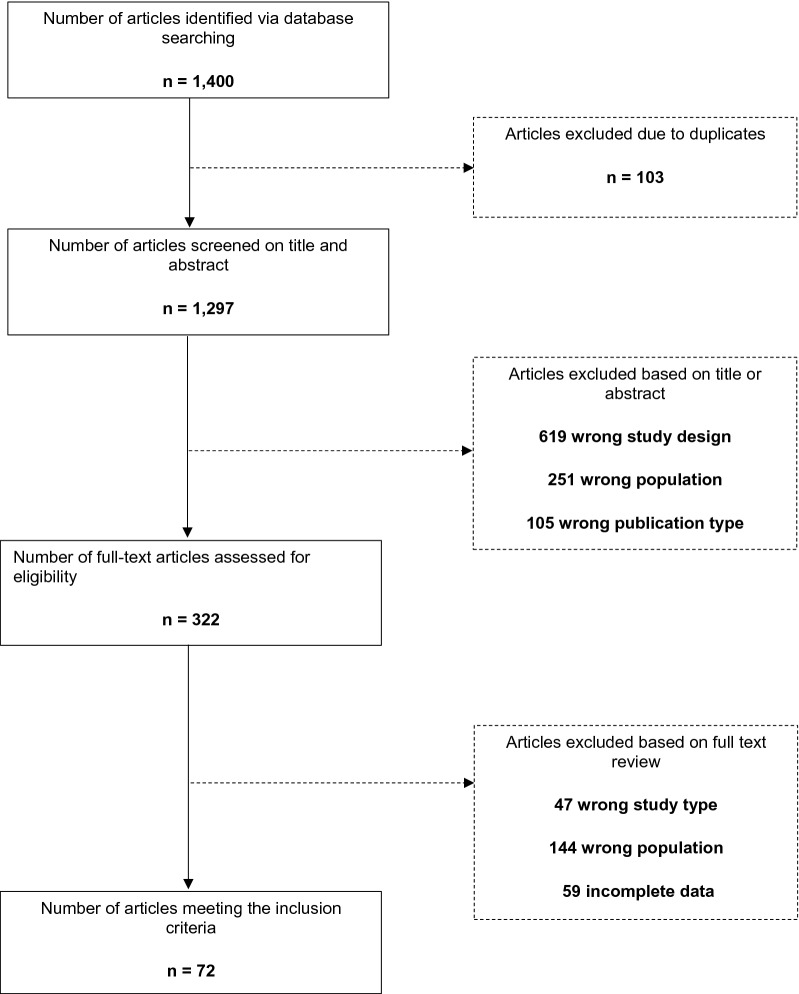
Flowchart of studies

A.N.G. and M.A.R. independently reviewed titles and abstracts considering eligibility criteria. Once the initial screening was completed, full-texts were reviewed by both researchers. Discrepancies in all steps were resolved by consensus. Figure 1 shows a PRISMA diagram depicting the study selection process.

### Data extraction

Two reviewers (G.L. and M.A.R.) independently extracted relevant data from the included studies using a standardized form and following a predetermined protocol. Extracted data included: title, first author, year of publication, language, study objective, study design, year of data collection, municipality and federative unit studied, sample size, gender, type of diabetes, duration of diabetes, skin color, mean age, frequency of DR, frequency by type of DR, classification of DR used, and diagnostic method of DR.

### Risk of bias assessment

Risk of Bias (RoB) was assessed in duplicate by L.P.S. and T.A.C., using a tool developed by Hoy et al. [27] for RoB estimation in prevalence studies. The tool comprises 10 items, classified as low or high risk of bias and a summary item that stratifies studies in low, moderate, or high risk of bias. Disagreements were resolved by discussion, with involvement of a third author when necessary. RoB plots were generated using the ‘robvis’ package for R (version 0.3.0) [28].

### Statistical analysis

Overall and subgroup prevalence estimates, with corresponding 95% confidence intervals, were estimated based on reported frequencies of DR in individuals with diabetes within the included studies. Pooled estimates were obtained by a random-effects inverse variance approach with arcsine transformation, assuming heterogeneity between studies due to the epidemiological nature of primary literature [29]. Confidence intervals for individual studies were estimated using the Clopper-Pearson approach. Percentage of total variability due to between-study heterogeneity was estimated by I^2^ statistic. Subgroup analyses were also performed to determine whether the following variables affected prevalence estimates: geographic region (South, Southeast, Midwest, North, and Northeast), diabetes duration (shorter than 10 and longer than 10 years), type of diabetes, year of the study (before or after 2000). All analyses were performed using the ‘meta’ package (version 6.0) for R (version 4.2.1). To reduce heterogeneity, potential factors that should affect it were explored, including studies that were carried out in ophthalmology services for the diagnosis of DR.

## Results

The search retrieved 1400 articles from October 1950 to February 2022, of which 103 were duplicates and were excluded. In total, 975 articles were removed based on title and abstracts; 322 full-text articles were assessed for eligibility, of which 72 met all inclusion criteria. Figure 1 shows the flowchart of study selection.

Table 1 shows the characteristics of the included studies by diagnostic criteria and method of assessment.

**Table 1:** Characteristics of the included studies

Study	Year of publication	Type of study	City, state, or region	Diabetes type	Diabetes duration Mean (± SD) or median (interquartile range)	n	Female (%)	Diagnostic method	Patients with retinopathy n, (%)	Patients with nonproliferative diabetic retinopathy, n, (%)	Patients with mild nonproliferative diabetic retinopathy n, (%)	Patients with ﻿moderate nonproliferative diabetic retinopathy n, (%)	Patients with severe nonproliferative diabetic retinopathy n, (%)	Patients with proliferative diabetic retinopathyn, (%)	Patients with diabetic macular edema n, (%)
Alvarenga et al. [30]	2003	Cross-sectional	São Paulo, SP			575	52.5	Ophthalmoscopy	293 (51)	185 (63.1)	90 (30.7)	71 (24.2)	24 (8.2)	108 (36.8)	
Andrade et al. [31]	2006	Cross-sectional	São Paulo, SP	Type 1 and 2		803	57.4	Ophthalmoscopy	399 (49.7)	267 (66.9)				132 (33.1)	80 (20)
Assis et al. [32]	2022	Cross-sectional	João Pessoa, PB	Type 2		285	34.7	Ophthalmoscopy	157 (55.1)	103 (65.6)				54 (34.4)	
Ben et al. [3]	2020	Cross-sectional	Porto Alegre, RS	Type 2		206	60.7	Ophthalmoscopy	49 (23.8)						
Cardoso et al. [33]	2012	Cross-sectional	Rio de Janeiro, RJ	Type 2		550	61.8		183 (33.3)						
Cardoso et al. [34]	2017	Cohort	Rio de Janeiro, RJ	Type 2		646			198 (30.7)	144 (72.7)				54 (27.3)	
Cardoso et al. [35]	2022	Cohort	Rio de Janeiro, RJ	Type 1 and 2	10 (± 8.3)	551			177 (32.1)						
Chacon et al. [36]	2005	Cross-sectional	Natal, RN	Type 1 and 2		76			42 (55.3)	30 (71.4)				12 (28.6)	
Chen-Ku et al. [37]	2019	Cohort		Type 2		437	53.1		23 (5.3)						
Costa e Silva et al. [38]	2018	Case–control	Porto Alegre, RS	Type 2	16 (± 8.1)	186	55.0	Color fundus photography	95 (51.1)	46 (48.4)				49 (51.6)	
Costa et al. [39]	2004	Cross-sectional	Porto Alegre and Passo Fundo, RS	Type 2		548		Ophthalmoscopy	221 (40.3)						
DePaula et al. [40]	2011	Cross-sectional	Goiânia, GO	Type 2	10.8 (± 5.9)	454	29.1		148 (32.6)						
Dieter et al. [41]	2022	Case–control	Porto Alegre, RS	Type 2		1005		Ophthalmoscopy	565 (56.2)						
Escarião et al. [19]	2008	Cross-sectional	Recife, PE	Type 1 and 2	8.1 (± 6.3)	2223	70.5	Ophthalmoscopy	566 (25.5)	492 (86.9)				74 (13.1)	66 (11.6)
Esteves et al. [42]	2009	Cross-sectional	Porto Alegre, RS	Type 1	14.4 (± 7.3)	437	49.7	Ophthalmoscopy	194 (44.4)	97 (50)	66 (34)	18 (9.3)	13 (6.7)	97 (50)	
Felício et al. [43]	2007	Cross-sectional	São Paulo, SP	Type 2	4 (0.5–38)	88	31.8	Ophthalmoscopy	29 (33)					2 (6.9)	4 (13.8)
Fernandez et al. [44]	1998	Cross-sectional	Uberlândia, MG	Type 1 and 2		605	66	Ophthalmoscopy	209 (34.5)	181 (86.6)				28 (13.4)	
Foss et al. [45]	1989	Cross-sectional	Ribeirão Preto, SP	Type 2	6 (0.1–43)	546	77.5	Ophthalmoscopy	129 (23.6)	107 (82.9)				22 (17.1)	
Galvão et al. [46]	2021	Cross-sectional	Goiânia, GO	Type 1 and 2		219	59.8	Ophthalmoscopy	70 (32)	53 (75.7)			18 (25.7)	17 (24.3)	21 (30)
Gomes et al. [47]	2021	Cross-sectional	Five regions: North, Northeast, Midwest, Southeast, South	Type 1	8.1 (± 4.3)	328		Ophthalmoscopy	28 (8.5)						
Gomes et al. [48]	1997	Cross-sectional	Rio de Janeiro, RJ	Type 1	7.5 (± 6.3)	50	56	Ophthalmoscopy	4 (8)						
Gomes et al. [49]	2009	Cross-sectional	Four regions: Northeast, Midwest, Southeast, South	Type 2	8.8 (± 7.2)	1382	59	Ophthalmoscopy	551 (39.9)						
Greca et al. [50]	2012	Cross-sectional	Porto Alegre, RS	Type 2		385		Ophthalmoscopy	251 (65.2)	108 (43)				143 (57)	
Gross et al. [51]	1993	Cross-sectional	Porto Alegre, RS	Type 2		117	49.6	Ophthalmoscopy	64 (54.7)						
Guedes et al. [52]	2009	Cross-sectional	Campos dos Goytacazes, RJ			46	78.3	Ophthalmoscopy	9 (19.6)	6 (66.7)				3 (33.3)	
Hirata et al. [53]	1986	Cross-sectional	São Paulo, SP	Type 1 and 2		138		Ophthalmoscopy	60 (43.5)	47 (78.3)				13 (21.7)	
Hissa et al. [54]	2002	Case–control	Fortaleza, CE	Type 2	10.5 (± 7.9)	44	72.7	Ophthalmoscopy	8 (18.2)						
Hokazono et al. [55]	2018	Cross-sectional	Curitiba, PR	Type 1 and 2	15 (0.3–40)	74	67.9	Ophthalmoscopy	50 (67.6)						
Jost et al. [56]	2010	Cross-sectional	Luzerna, SC	Type 2		120	51.7	Ophthalmoscopy	46 (38.3)	41 (89.1)				5 (10.9)	
Junior et al. [57]	2001	Cross-sectional	São Paulo, SP	Type 1 and 2	12.7 (± 5.7)	103	44.7	Ophthalmoscopy	74 (71.8)	43 (58.1)				31 (41.9)	
Kramer et al. [58]	2011	Cross-sectional	Porto Alegre, RS	Type 2		207	46.9	Ophthalmoscopy	83 (40.1)						
Kramer et al. [59]	2009	Cross-sectional	Porto Alegre, RS	Type 2		65		Ophthalmoscopy	14 (21.5)						
Kramer et al. [60]	2008	Cohort	Porto Alegre, RS	Type 1 and 2		112	42.9	Ophthalmoscopy	43 (38.4)						
Lima et al. [61]	2018	Cross-sectional	Volta Grande, MT	Type 2		140	71.4	Ophthalmoscopy	27 (19.3)						
Lima et al. [62]	2021	Cross-sectional	Porto Alegre, RS	Type 1 and 2	10 (2–34)	70	60	Ophthalmoscopy	27 (38.6)						
Malerbi et al. [63]	2020	Cross-sectional	Volta Grande, MT	Type 2		95	66.3	Color fundus photography	16 (16.8)	15 (93.8)	2 (12.5)	12 (75)	1 (6.2)	1 (6.2)	6 (37.5)
Malerbi et al. [64]	2021	Cross-sectional	Itabuna, BA	Type 2		366	60.1	Color fundus photography	93 (25.4)	68 (73.1)				25 (26.9)	29 (31.2)
Malerbi et al. [65]	2022	Cross-sectional	Itabuna, BA	Type 2	10.4 (± 8.7)	824		Color fundus photography	253 (30.7)	181 (71.5)	75 (29.6)	71 (28)	35 (13.8)	72 (28.5)	
Martins et al. [66]	2004	Case–control	São Paulo, SP			103		Ophthalmoscopy	74 (71.8)	60 (81.1)				14 (18.9)	
Massaro et al. [67]	2019	Cross-sectional	São Paulo, SP	Type 1 and 2		161	64	Ophthalmoscopy	31 (19.3)						
Massignam et al. [68]	2021	Case–control	Porto Alegre, RS	Type 1		410		Ophthalmoscopy	195 (47.6)	48 (24.6)				147 (75.4)	
Mata et al. [69]	2016	Cross-sectional	Belo Horizonte, MG	Type 1 and 2		343	67.9		122 (35.6)						
Melo et al. [20]	2018	Cross-sectional	Five regions: North, Northeast, Midwest, Southeast, South	Type 1	15.3 (± 9.3)	1644	55.8	Ophthalmoscopy	589 (35.8)	417 (70.8)	298 (50.6)	108 (18.3)	11 (1.8)	172 (29.2)	44 (7.4)
Mori et al. [70]	2021	Cross-sectional	São Paulo, SP	Type 1		405		Color fundus photography	271 (66.9)						
Mota et al. [71]	2011	Cross-sectional	Fortaleza, CE	Type 2	8.1 (± 6.5)	145	57.9		26 (17.9)	21 (80.8)				5 (19.2)	
Parisi et al. [72]	2016	Cross-sectional	Four regions: Northeast, Midwest, Southeast, South	Type 1 and 2	14.2 (± 9.8)	1055	58.6	Ophthalmoscopy	487 (46.2)						
Pedrosa et al. [73]	2012	Cross-sectional	Ananindeua, PA		11.8	31	54.8	Ophthalmoscopy	11 (35.5)	11 (100)					
Penido et al. [74]	2001	Cross-sectional	Conselheiro Lafaiete, MG	Type 1 and 2		314	69.1	Ophthalmoscopy	132 (42)						
Piccirillo et al. [75]	2002	Cross-sectional	Rio de Janeiro, RJ	Type 1		71		Ophthalmoscopy	11 (15.5)						
Polina et al. [76]	2019	Case–control	Porto Alegre, RS	Type 2	14.7 (± 7.9)	546	54	Ophthalmoscopy and color fundus photography	302 (55.3)	161 (53.3)				141 (46.7)	
Preti et al. [77]	2010	Cross-sectional	São Paulo, SP	Type 2		105	54.3	Ophthalmoscopy	90 (85.7)	53 (58.9)	30 (33.3)	18 (20)	5 (5.5)	37 (41.1)	
Queiroz et al. [16]	2020	Cross-sectional	São Paulo, SP	Type 2	10.7 (± 8.2)	627	62.4	Color fundus photography	106 (16.9)						
Rodrigues et al. [78]	2010	Cross-sectional	Porto Alegre, RS			441		Ophthalmoscopy	197 (44.7)	97 (49.2)	66 (33.5)	18 (9.1)	13 (6.6)	100 (50.7)	
Rodrigues et al. [79]	2015	Cross-sectional	Belo Horizonte, MG	Type 2		102	81.4	Ophthalmoscopy	66 (64.7)						
Rosa et al. [80]	2019	Cohort	Rio de Janeiro, RJ	Type 1		167		Ophthalmoscopy	30 (18)						
Rosses et al. [81]	2017	Cross-sectional	Porto Alegre, RS	Type 2	6 (3–14)	219	59.8	Color fundus photography	39 (17.8)	36 (92.3)	7 (17.9)	24 (61.5)	5 (12.8)	3 (7.7)	
Sampaio et al. [82]	2007	Cross-sectional	Londrina, PR	Type 1	13.4 (± 5.8)	81	64.2	Ophthalmoscopy	17 (21)	7 (41.2)				10 (58.8)	
Santos et al. [83]	2005	Cross-sectional	Porto Alegre, RS	Type 2	10.5 (± 9.7)	210	67.9	Ophthalmoscopy	99 (47.1)						
Santos et al. [84]	2005	Case–control	Rio Grande do Sul	Type 2		477	55.6	Ophthalmoscopy	275 (57.7)						
Scheffel et al. [85]	2008	Cross-sectional	Porto Alegre, Rio Grande and Passo Fundo, RS	Type 2		1182	51.4	Ophthalmoscopy	508 (43)						
Schellini et al. [22]	2014	Cross-sectional	São Paulo	Type 2		407	67.8	Ophthalmoscopy	31 (7.6)						
Schmid et al. [86]	1995	Cross-sectional	Porto Alegre, RS	Type 2		35		Ophthalmoscopy	19 (54.3)	10 (52.6)				9 (47.4)	
Serfaty et al. [87]	2010	Cross-sectional	Rio de Janeiro, RJ	Type 1		47		Ophthalmoscopy	7 (14.9)	7 (100)					
Souza et al. [18]	2004	Cross-sectional	Ribeirão Preto, SP	Type 1 and 2		2360	65	Ophthalmoscopy	478 (20.3)						
Souza et al. [21]	2020	Cross-sectional	Viçosa and Santo Antônio do Monte, MG			1331	62.8	Color fundus photography	394 (29.6)						
Steck et al. [88]	1993	Cross-sectional	Franco da Rocha, SP	Type 1 and 2		142	66.2	Ophthalmoscopy	104 (73.2)					21 (20.2)	
Sugano et al. [89]	2001	Cross-sectional	São Bernardo do Campo, SP	Type 1 and 2		215	47.9	Ophthalmoscopy	53 (24.7)						
Syllos et al. [90]	2006	Case–control	Passo Fundo, RS	Type 2	15.9 (± 5.7)	170	60	Ophthalmoscopy	56 (32.9)						
Tres et al. [91]	2007	Cross-sectional	Passo Fundo, RS	Type 2	8 (± 6.7)	340	59.7	Ophthalmoscopy	98 (28.8)						
Viégas et al. [92]	2011	Cross-sectional	Recife, PE	Type 2	10.25 (± 8.5)	148	100	Ophthalmoscopy	51 (34.5)						
Wobeto et al. [93]	2007	Cross-sectional	Campinas, SP	Type 1 and 2		317	62.1	Ophthalmoscopy	156 (49.2)						
Wolosker et al. [94]	1995	Cross-sectional	São Paulo, SP	Type 1 and 2		70	60		14 (20)						

**Fig. 2:**
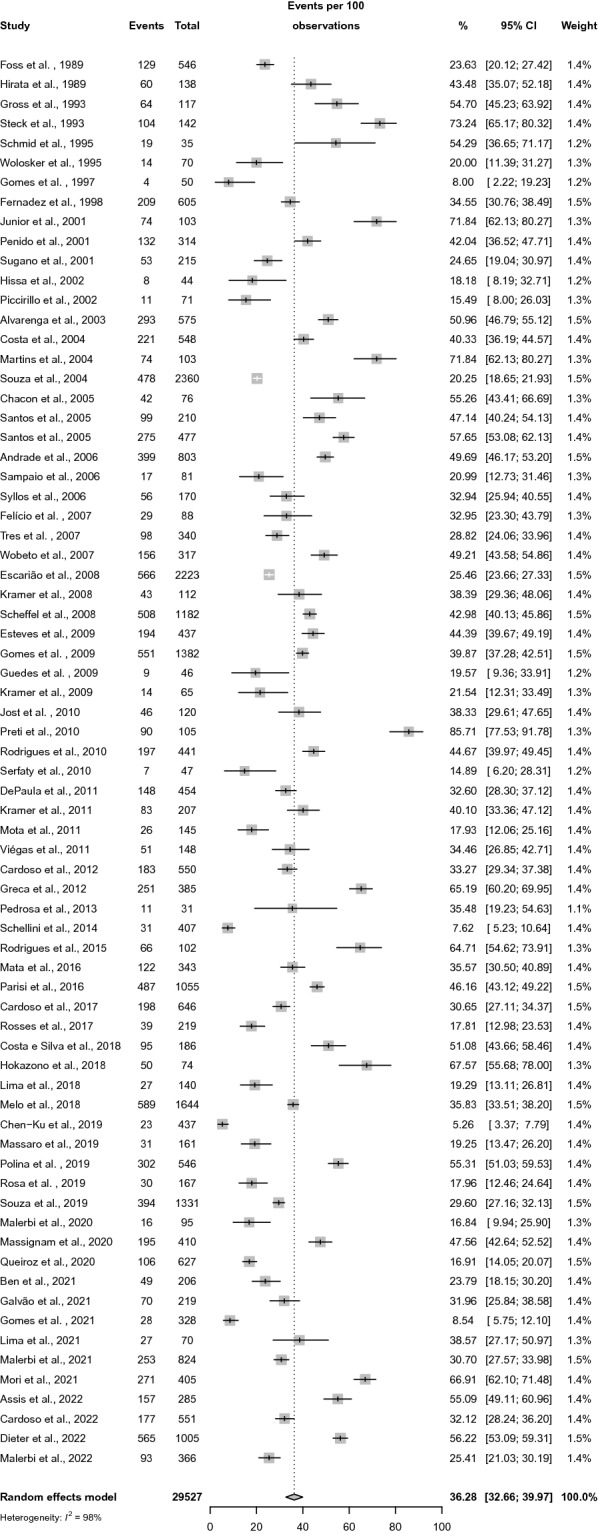
Forest plot representing diabetic retinopathy prevalence rates

A meta-analysis was conducted; Fig. 2 shows prevalence rates. The prevalence rate of DR (pooled estimate) was 36.28% (95% CI 32.66–39.97, I^2^ 98%).

We could not assess the prevalence of DR by gender because such variable was unspecified in most studies.

Trend analyses showed an increase in the prevalence of DR in patients with longer duration of diabetes [20.73% (95% CI 11.63–31.64 I^2^ 98%) in less than 10 years of diabetes, and 37.73% (95% CI 30.80–44.93 I^2^ 96%) in longer than 10 years] (Additional file 2: Fig. S1) and in patients with type 2 diabetes mellitus [26.84% (95% CI 16.43–38.74, I^2^ 98%) in type 1 diabetes mellitus and 35.69% (95% CI 30.16–41.41, I^2^ 98%) in type 2 diabetes mellitus] (Additional file 3: Fig. S2).

In the assessment of prevalence of DR according to the year of publication of the study, no difference in the prevalence of DR [36.06% (95% CI 31.59–40.65, I^2^ 98%) was observed in articles published after 2000 and 37.84% (95% CI 22.90–54.07, I^2^ 96%) in those published before 2000] (Additional file 4: Fig. S3).

The analysis of prevalence rates by diagnostic method showed a prevalence of 38.15% (95% CI 33.08–43.36, I^2^ 98%) in patients diagnosed by indirect ophthalmoscopy, and 31.11% (95% CI 19.55–44.00, I^2^ 98%) by color fundus photography (CFP, Additional file 5: Fig. S4).

We explored potential factors that would affect the heterogeneity of the analyses, including studies that were carried out in ophthalmology services for the diagnosis of DR. Table 2 shows prevalence rates of DR and their 95% CI by Brazilian regions and adjustment to studies that were not performed in ophthalmology services. The data in this table show that the prevalence of DR was higher in the Southern region. No significant change of the heterogeneity was observed when we did this type of new analyses.

**Table 2:** Prevalence of diabetic retinopathy by region; analyses with and without articles done in reference centers

Region	All articles		Analyses without articles that were not done in ophthalmology centers	
	% (95% CI)	I^2^%	% (95% CI)	I^2^%
Midwest	25.38 [17.72–33.90]	84	23.03 [13.33–34.45]	88
Northeast	32.25 [22.73–42.58]	95	30.21 [20.76–40.59]	95
North	35.48 [19.23–54.63]	–	35.48 [19.23–54.63]	–
Southeast	35.96 [28.18–44.12]	98	30.11 [24.27–36.29]	97
South	42.68 [37.13–48.33]	94	41.33 [36.28–46.46]	95

### Quality of studies

**Fig. 3:**
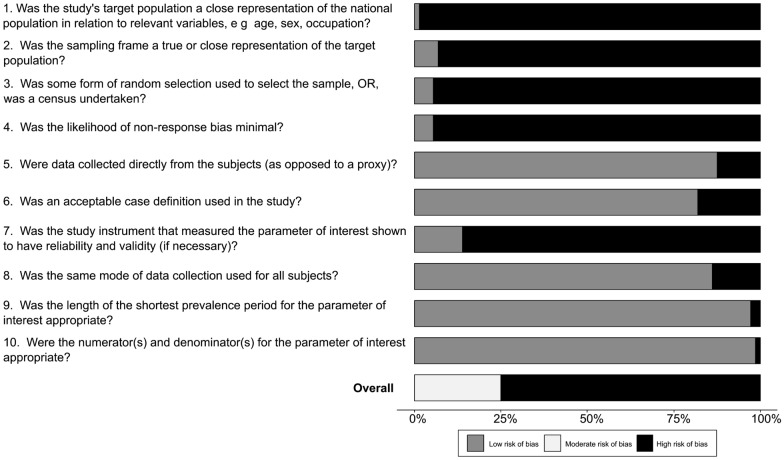
Quality of studies characteristics

Figure 3 summarizes data regarding quality of studies. A total of 18 studies (25%) had an intermediate risk of bias, and the rest of the studies had a high risk of bias. Additional file 6: Fig. S5 shows the risk of bias assessment for each study. Most studies were based on cross-sectional design (59 studies, 82%). The most used design was convenience sampling (68 studies, 94%). Most studies were developed only in or including data from Southeastern and Southern Brazil (30 studies, 48.4% and 24 studies, 33.3%, respectively). In 23 studies (31.9%), the main objective was to evaluate the prevalence of DR.

## Discussion

Our study provides comprehensive and up-to-date evaluations of the current DR prevalence in Brazil with the largest meta-analysis to date.

This study included 72 studies carried out in Brazil and found a 36.26% prevalence of DR. It also found a higher prevalence in patients with long-term disease, type 2 diabetes, and residents of the Southern region.

According to the IDF 2021 atlas [15], 15.7 million people live with diabetes in Brazil, being the sixth country with the highest number of people with diabetes. Teló et al.—in a systematic review with meta-analysis of Brazilian observational studies from 1980 to 2015—included 50 studies and showed an increasing prevalence of diabetes in recent decades, showing that the prevalence of diabetes in Brazil can reach 6.9% of the population in studies published after 2010 [6]. Data obtained from the National Survey of Health (2014 to 2015) showed the following prevalence of diabetes according to different criteria: 6.6% (95% CI 5.9–7.2) [glycated hemoglobin (HbA1c) ≥ 6.5% (47.5 mmol/mol)]; 8.4% (95% CI 7.6–9.1) [HbA1c ≥ 6.5% (47.5 mmol/mol) or use of antidiabetic drugs]; 9.4% (95% CI 8.6–10.1) [HbA1c ≥ 6.5% (47.5 mmol/mol) or history of diabetes]; and 7.5% (95% CI 6.7–to 8.2) [history of diabetes] [95]. Extrapolating the prevalence of DR found in our review, 5.7 million people would be living with DR in Brazil. Comparing our outcomes with other systematic reviews that evaluated the prevalence of DR, our study showed a higher prevalence than China (18.45%) [96], Africa (30.2–31.6%) [97], and Europe (25.7%) [98]. In India, a systematic review performed with 8,866 diabetic patients found a 16.1% prevalence of RD [11, 98]; in Pakistan, a systematic review estimated the prevalence of DR at 28.2%, ranging from 10.6 to 91.34% [12]; in the USA, a study carried out from 2005 to 2008 with 1495 diabetic patients showed a 47% prevalence of DR [13]; in Indonesia, a 2017 study showed a DR prevalence of 43.1% in patients with type 2 diabetes mellitus [14]. Different study methodologies applied in primary studies retrieved in these different meta-analyses are important to determine these high different figures among countries.

Regarding the predictors evaluated in trend analysis, the duration of diabetes increases the prevalence of DR. Duration of diabetes is an established risk factor for the development of DR and other microvascular complications in patients with diabetes [99–101]. The same trend was described in another systematic review [14].

The study showed a higher prevalence of DR in type 2 diabetes mellitus patients, but the literature shows that prevalence is higher in type 1 diabetes mellitus [7]. One of the possibilities is that the studies on patients with type 1 diabetes mellitus have been carried out with a short duration of disease. Moreover, since type 2 diabetes mellitus is more prevalent than type 1 diabetes mellitus, a higher number of people with type 2 diabetes mellitus are possibly being studied as compared to those with type 1 diabetes mellitus, unbalancing the final outcomes.

This meta-analysis showed that the prevalence of DR in the Southern region is higher than in the Northern region. This can be explained because disease burden components have different distributions between the North and the South in Brazil, due to economic and social disparities between regions. As a country with continental territory, racial and cultural miscegenation, Brazil experiences great social and economic problems, including socioeconomic inequality. The Federation Unit with the lowest poverty rate in 2021 was Santa Catarina (10.16%) in the South and the one with the highest proportion of poor people was Maranhão with 57.90% in the Northeast. Segmenting the country into 146 spatial strata, the one with the greatest poverty in 2021 is the Coast and Baixada Maranhense with 72.59%, while the lowest is in the municipality of Florianópolis in South with 5.7% [102]. It is theorized that patients living in the Northern region do not live long enough to develop microvascular complications of diabetes [103] and, as aforementioned, the duration of the disease is one of the markers that increase the risk of developing DR. Another difference may be because in the Northern region access to public health is more limited [104].

The gold standard method for screening for DR is CFPs [105]. In this review, eight (11%) studies used CFPs as a diagnostic method and 53 (73.6%) used ophthalmoscopy. Ophthalmoscopy and color fundus photographs are both valid strategies for DR screening. Each method has advantages and potential limitations that include cost, expertise of the examiners, and equipment. The main advantages of ophthalmoscopy are its easy handling and superior performance in cases of poor patient collaboration, on the other hand, it is not sensitive enough to detect minor signs of DR and depends on the presence of a trained operator [106]. In turn, CFPs has the advantage of providing a permanent record of retinopathy, which can be used later to document retinopathy progression and allows for a more detailed grading of retinopathy. Notably, CFPs are expensive [107].

Some studies were carried out in ophthalmology services for the diagnosis of DR, which may represent a selection bias, considering that reference services will likely have a higher rate of patients with complications. Therefore, we performed an analysis excluding the studies conducted in ophthalmology services as shown in Table 2, resulting in a decrease in the prevalence of DR.

Our study has some limitations, the most important being the high heterogeneity. Migliavaca et al. show that prevalence studies have high heterogeneity [108]. The available smaller studies conducted in Brazil are often limited in scope and may uncover confounding or conflicting results due to their small sample size. The heterogeneous nature of studies (e.g., patient selection criteria, diabetes type, setting), disparity between study methods (color fundus photography vs*.* ophthalmoscopy), and possible differences between urban and rural settings, with variations on the following items: access to healthcare and eating habits [109, 110] may contribute to conflicting reports of prevalence and incidence, making a direct comparison of studies difficult. Most studies did not report visual acuity or the prevalence of maculopathy or proliferative DR, both considered vision-threatening DR. Although the significance of functional outcomes, most epidemiological studies do not address them. Rates of blindness are variable among countries (high-income vs. low- to middle-income countries) depending on the existence of screening programs, specialized workforce, and possibility of timely treatment [111]. Such limitations highlight the need for consistent data capture in Brazil.

## Conclusion

This study shows a high heterogeneity that is expected in systematic reviews estimating prevalence rates. It is necessary to develop multicenter studies with representative samples and standardized methodology. Screening programs are effective for the identification of early DR, and epidemiological studies are essential for their success, as they collect data that allows identification of the magnitude of the problem, as well as regional differences. Further research is needed to collect such data in Brazil, with the use of standardized criteria and consistent terminology, and the inclusion of samples that are representative of the communities from which they are drawn. Robust longitudinal collection of patient data will be essential to allow identification of the true extent of diagnosed retinal complications of diabetes, in turn providing healthcare planners with essential information to aid future decision-making.

## Supplementary Information

**Additional file 1:** **Table S1.** Literature search strategy used.

**Additional file 2.** **Figure S1.** Forest plot representing diabetic retinopathy prevalence rates by duration of diabetes.

**Additional file 3.** **Figure S2.** Forest plot representing diabetic retinopathy prevalence rates by diabetes type.

**Additional file 4.** **Figure S3.** Forest plot representing diabetic retinopathy prevalence rates by study publication year.

**Additional file 5.** **Figure S4.** Forest plot representing diabetic retinopathy prevalence rates by diagnostic method.

**Additional file 6.** **Figure S5.** Risk of bias assessment in the included studies.

## Data Availability

The datasets supporting the conclusions of this article are included within the article and in the Additional files.

## Abbreviations

DR: Diabetic retinopathy
RoB: Risk of Bias
HbA1c: Glycated hemoglobin
CFP: Color fundus photography
