# Supplementary material for: Prevalence of diabetic retinopathy in Brazil: a systematic review with meta-analysis
Source: Diabetol Metab Syndr. 2023 Mar 2;15:34. doi: 10.1186/s13098-023-01003-2 (PMC9979496; doi:10.1186/s13098-023-01003-2)
Supplement: Supplementary file 1 — Additional file 1: Table S1. Literature search strategy used. [file 13098_2023_1003_MOESM1_ESM.docx]

**Table S1. Literature Search Strategy Used**

| **PUBMED**  **#1** Search: ("Retinal Diseases"[Mesh]) OR "Diabetic Retinopathy"[Mesh] OR "diabetic retinopathy" OR retinopathy [Title/Abstract]  **#2** Search: Brazil [Mesh] OR Brazil* [Title/Abstract] OR Brazil [Title/Abstract] OR "Minas Gerais" [Title/Abstract] OR "São Paulo" [Title/Abstract] OR "Espirito Santo" [Title/Abstract] OR "Rio de Janeiro" [Title/Abstract] OR Bahia [Title/Abstract] OR Pará [Title/Abstract] OR "Mato Grosso" [Title/Abstract] OR "Mato Grosso do Sul" [Title/Abstract] OR Goiás [Title/Abstract] OR "Rio Grande do Sul" [Title/Abstract] OR Ceará [Title/Abstract] OR Pernanbuco [Title/Abstract] OR "Santa Catarina" [Title/Abstract] OR Amazonas [Title/Abstract] OR Maranhão [Title/Abstract] OR Tocantins [Title/Abstract] OR Piauí [Title/Abstract] OR Rondônia [Title/Abstract] OR Roraima [Title/Abstract] OR Paraná [Title/Abstract] OR Acre [Title/Abstract] OR Amapá [Title/Abstract] OR Paraíba [Title/Abstract] OR "Rio Grande do Norte" [Title/Abstract] OR Alagoas [Title/Abstract] OR Sergipe [Title/Abstract] OR "Distrito Federal" [Title/Abstract]  **#3** Search: #1 AND #2  **LILACS**  "Retinal Diseases" OR "Diabetic Retinopathy" OR "diabetic retinopathy" OR retinopathy [Palavras] and Brazil OR Brazil OR "Minas Gerais" OR "São Paulo" OR "Espirito Santo" OR "Rio de Janeiro" OR Bahia OR Pará OR "Mato Grosso" OR "Mato Grosso do Sul" OR Goiás OR "Rio Grande do Sul" OR Ceará OR Pernanbuco OR "Santa Catarina" OR Amazonas OR Maranhão OR Tocantins OR Piauí OR Rondônia OR Roraima OR Paraná OR Acre OR Amapá OR Paraíba OR "Rio Grande do Norte" OR Alagoas OR Sergipe OR "Distrito Federal" [Palavras] and prevalence [Palavras]  **EMBASE**  'diabetes AND mellitus'/exp AND 'retinopathy'/exp AND 'prevalence'/exp AND 'observational AND study'/exp |
| --- |
