# Supplementary figures and images for: Prevalence of diabetic retinopathy in Brazil: a systematic review with meta-analysis
Source: Diabetol Metab Syndr. 2023 Mar 2;15:34. doi: 10.1186/s13098-023-01003-2 (PMC9979496; doi:10.1186/s13098-023-01003-2)

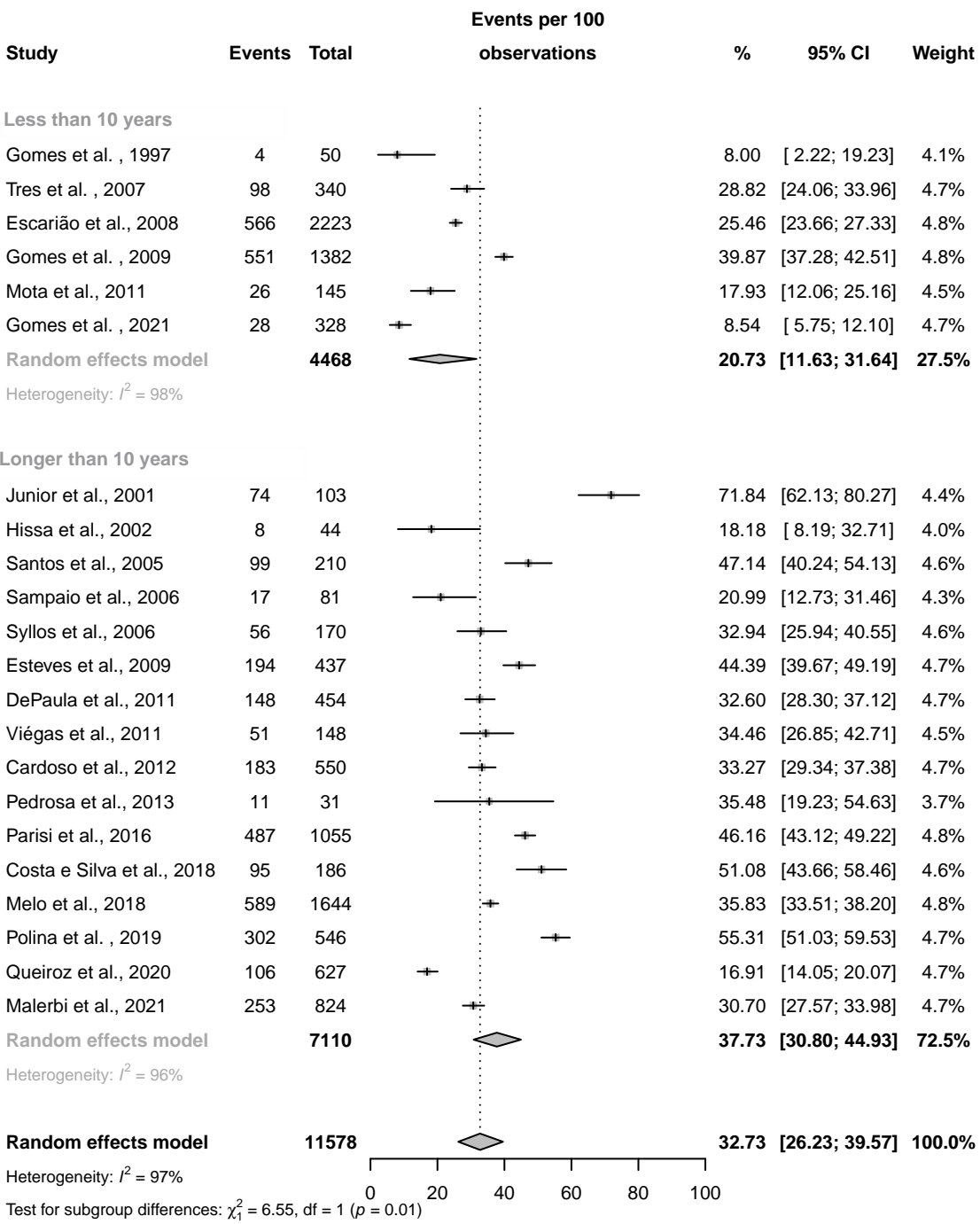

Supplement: Supplementary file 2 — Additional file 2. Figure S1. Forest plot representing diabetic retinopathy prevalence rates by duration of diabetes. [file 13098_2023_1003_MOESM2_ESM.pdf]

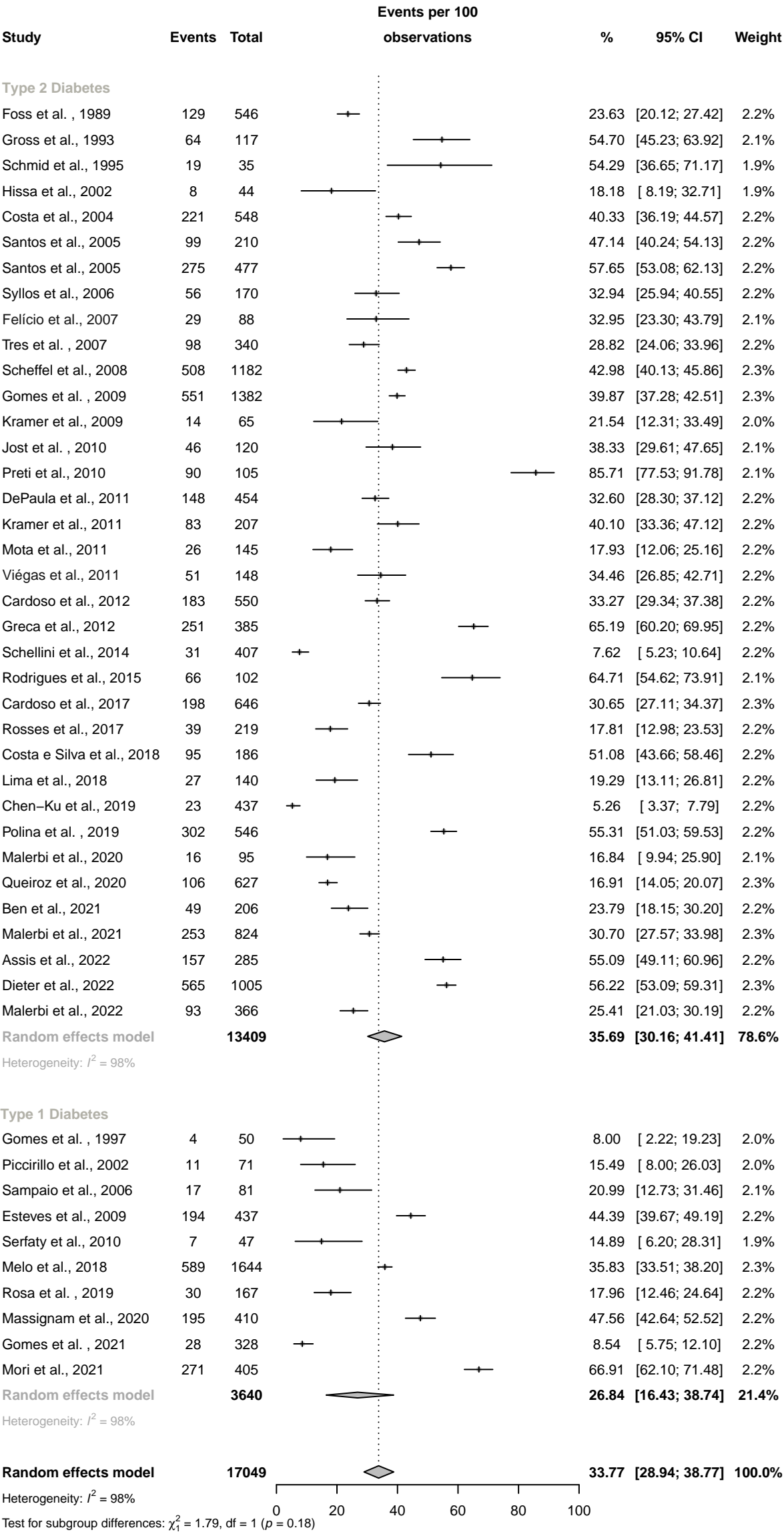

Supplement: Supplementary file 3 — Additional file 3. Figure S2. Forest plot representing diabetic retinopathy prevalence rates by diabetes type. [file 13098_2023_1003_MOESM3_ESM.pdf]

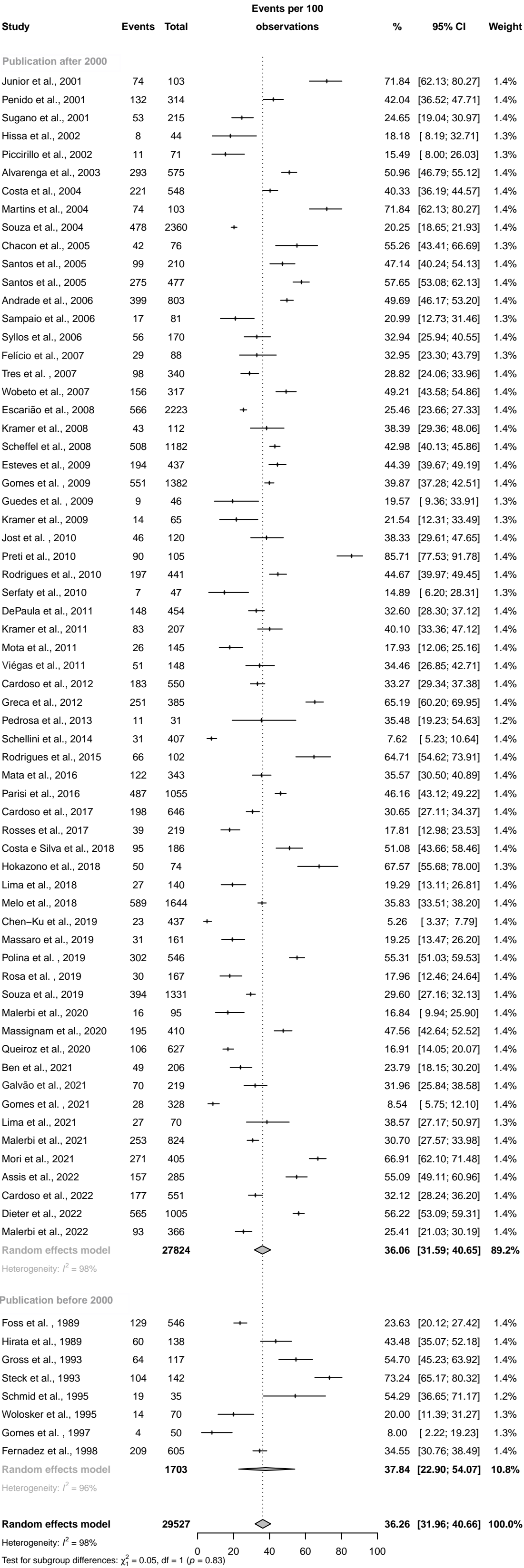

Supplement: Supplementary file 4 — Additional file 4. Figure S3. Forest plot representing diabetic retinopathy prevalence rates by study publication year. [file 13098_2023_1003_MOESM4_ESM.pdf]

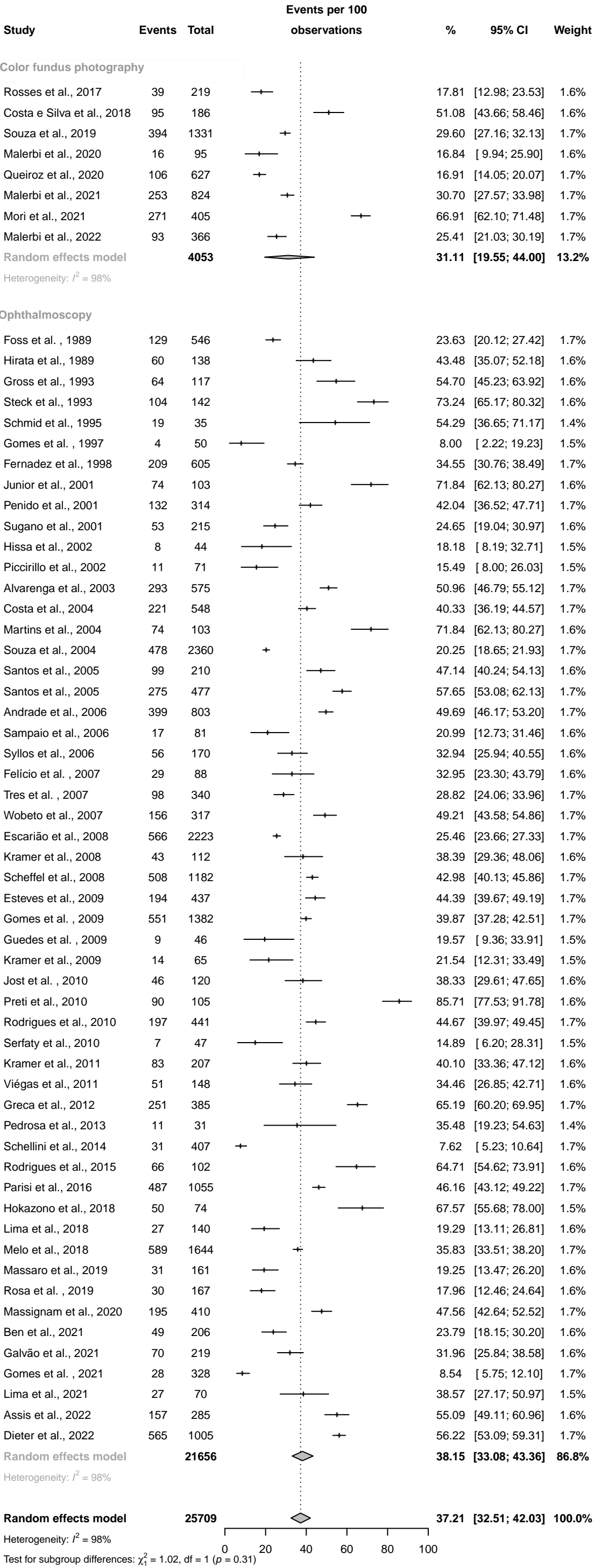

Supplement: Supplementary file 5 — Additional file 5. Figure S4. Forest plot representing diabetic retinopathy prevalence rates by diagnostic method. [file 13098_2023_1003_MOESM5_ESM.pdf]
